# Early pregnancy loss in 15-hydroxyprostaglandin dehydrogenase knockout (15-HPGD^−/−^) mice due to requirement for embryo 15-HPGD activity

**DOI:** 10.1038/s41598-019-54064-7

**Published:** 2019-11-26

**Authors:** Jeffrey D. Roizen, Minoru Asada, Min Tong, Hsin-Hsiung Tai, Louis J. Muglia

**Affiliations:** 1 Division of Endocrinology and Diabetes (JDR), and, The Children’s Hospital of Philadelphia, Philadelphia, PA, and University of Pennsylvania Perelman School of Medicine, Philadelphia, PA United States 19104; 2 Osaka Shiritsu Sogo Iryo Center, Osaka, Japan; 3 Department of Pharmaceutical Sciences (MT, HHT), College of Pharmacy, University of Kentucky, Lexington, KY 40536 USA; 4 Center for Prevention of Preterm Birth (LJM), Cincinnati Children’s Hospital Medical Center, Cincinnati, OH 45229 USA

**Keywords:** Embryology, Endocrine reproductive disorders

## Abstract

Prostaglandins (PGs) have critical signaling functions in a variety of processes including the establishment and maintenance of pregnancy, and the initiation of labor. Most PGs are non-enzymatically degraded, however, the two PGs most prominently implicated in the termination of pregnancy, including the initiation of labor, prostaglandin E2 (PGE_2_) and prostaglandin F2α (PGF_2α_), are enzymatically degraded by 15-hydroxyprostaglandin dehydrogenase (15-HPGD). The role of PG metabolism by 15-HPGD in the maintenance of pregnancy remains largely unknown, as direct functional studies are lacking. To test the hypothesis that 15-PGDH-mediated PG metabolism is essential for pregnancy maintenance and normal labor timing, we generated and analyzed pregnancy in 15-HPGD knockout mice (Hpgd^−/−^). We report here that pregnancies resulting from matings between 15-HPGD KO mice (*Hpgd*^−/−^ X *Hpgd*^−/−^KO mating) are terminated at mid gestation due to a requirement for embryo derived 15-HPGD. Aside from altered implantation site spacing, pregnancies from KO matings look grossly and histologically normal at days post coitum (dpc) 6.5 and 7.5 of pregnancy. However, virtually all of these pregnancies are resorbed by dpc 8.5. This resorption is preceded by elevation of PGF_2∝_ but is not preceded by a decrease in circulating progesterone, suggesting that pregnancy loss is a local inflammatory phenomenon rather than a centrally mediated phenomena. This pregnancy loss can be temporarily deferred by indomethacin treatment, but treated pregnancies are not maintained to term and indomethacin treatment increases maternal mortality. We conclude that PG metabolism to inactive products by embryo derived 15-HPGD is essential for pregnancy maintenance in mice, and may serve a similar function during human pregnancy.

## Introduction

Prostaglandins (PGs) have critical signaling functions in a variety of processes including reproduction, blood pressure homeostasis, sexual dimorphism and inflammation^1–5^. PGE_2_ and PGF_2α_, have critical signaling functions in determining the termination of labor at term and prior to term in humans and mice^6–8^. 15-hydroxyprostaglandin dehydrogenase (15-HPGD) is the enzyme primarily responsible for the enzymatic metabolism of PGE_2_ and PGF_2α_ to inactive products^9,10^. A great deal of research has examined the role of PG synthesis enzymes in PG signaling, giving rise to the paradigm of tight control of PG generation through regulation of the expression of PG synthesis enzymes. Of particular interest, some researchers have shown the coordinate regulation of the expression or activity of phospholipaseA2, the Cox enzymes and later prostaglandin synthetases in response to a variety of stimuli^11^.

During gestation, 15-HPGD levels in the placenta and fetal membranes increase as gestation nears term^12^. In a mouse model of bacterial-induced preterm labor and pregnancy termination, where intrauterine injection of heat killed *Escherichia coli* (HKE) induces early labor, one group has observed an increase in Cox-2 mRNA in the myometrium concurrent with a decrease in 15-HPGD mRNA in the fetal membranes and the fetus^13^. In this model, the induction of preterm labor correlated inversely with the relative abundance of 15-HPGD after HKE injection and not with any alteration in the abundance of Cox-2. In human parturition, placental and fetal membrane 15-HPGD mRNA and activity are lower in spontaneously laboring women at term than in non-laboring women at term^14,15^. In addition, expression of 15-HPGD is decreased further in chorion trophoblast cells of women with idiopathic preterm labor^14^. In determining the timing of labor, the roles of synthesis pathways leading to increased levels of PGF_2α_ and of the signaling pathways following PGF_2α_ receptor activation have characterized in great detail^8,16–20^. However, the role of PG metabolism to inactive products by 15-HPGD in the context of pregnancy termination remains largely unknown. The observations described above suggest that in both mouse and human parturition, regulation of 15-HPGD activity may play an integral role in determining the duration of pregnancy through control of PG metabolism to inactive products. To test the hypothesis that 15-HPGD mediated PG metabolism to inactive products regulates pregnancy length, we generated and analyzed 15-HPGD knockout (*Hpgd*^−/−^) mice.

## Results

**Figure 1:**
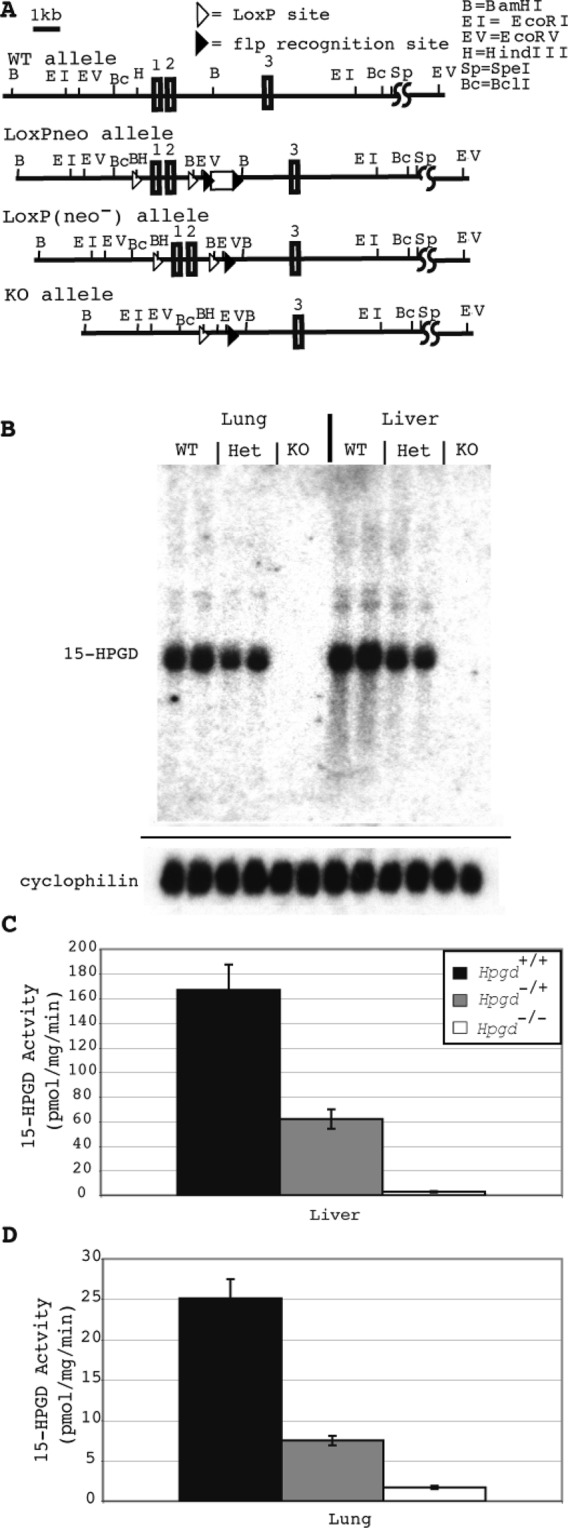
15-HPGD knockout allele completely abrogates 15-HPGD mRNA gene expression and 15-HPGD activity. (**A**) Wild-type *Hpgd*^+/+^, LoxPneo (targeting construct), LoxP(neo^−^) and Knockout *Hpgd*^−/−^ alleles. (**B**) *Hpgd*^−/−^ mice exhibit a complete absence of 15-HPGD mRNA as analyzed using northern blot of 15-HPGD mRNA in the lung and liver. Cyclophilin mRNA detection is provided as loading control, n = 2 for each genotype. The entire 15-HPGD blot is provided, whilc the cyclophilin blot is truncated for space as the demonstrated differences are qualitative or binary (i.e. positive or negative) rather than quantitative. (**C**) *Hpgd*^−/−^ mice completely lack 15-HPGD activity in the liver. Values are mean ± s.e.m. n = 3. (**D**) *Hpgd*^−/−^ mice completely lack 15-HPGD activity in the lung. Values are mean ± s.e.m. n = 3.

We generated mice homozygous for a 15-HPGD null (knockout) allele (Fig. 1A, Knockout Allele, *Hpgd*^−/−^). To confirm that our genetic effect had the desired effect of completely abrogating *Hgpd* gene transcription and activity, we examined 15-HPGD mRNA by Northern blot (Fig. 1B) and performed activity assays (Fig. 1C,D) in the lung and liver. 15-HPGD mRNA abundance was measured in the tissues expressing the highest levels of this enzyme: the liver and lung^10^. In both of these tissues, *Hpgd*^−/−^ mice have a complete absence of 15-HGPD mRNA and a complete absence of prostaglandin dehydrogenase activity (Fig. 1B–D).

At birth, litters from *Hpgd* heterozygote matings (*Hpgd*^+/−^ X *Hpgd*^+/−^) contain normal Mendelian ratios of knockout mice (*Hpgd*^−/−^), to heterozygotes (*Hpgd*^+/−^) to wild-types (*Hpgd*^+/+^ Table 1). However, all *Hpgd*^−/−^ mice fail to survive to week two of life. Other workers have reported that neonatal death in *Hpgd*^−/−^ mice is due to a patent ductus arteriosus and is rescued by neonatal injection of indomethacin^21^. After neonatal injection of indomethacin, *Hpgd*^−/−^ mice in litters from *Hpgd* heterozygote matings survive to adulthood with no overt phenotype.

**Table 1:** Fetal Neonatal Death and Rescue by Indomethacin injection in *Hpgd*^−/−^ mice.

Genotype	*Hpgd*^+/+^	*Hpgd*^−/+^	*Hpgd*^−/−^	*χ*^2^
Time of genotyping				
At Birth - no indomethacin injection)	10	16	10	>0.25
To day 14 - no Indomethacin injection	14	25	0	** <0.01
To day 14 - with Indomethacin Injection	12	19	8	>0.25

**Figure 2:**
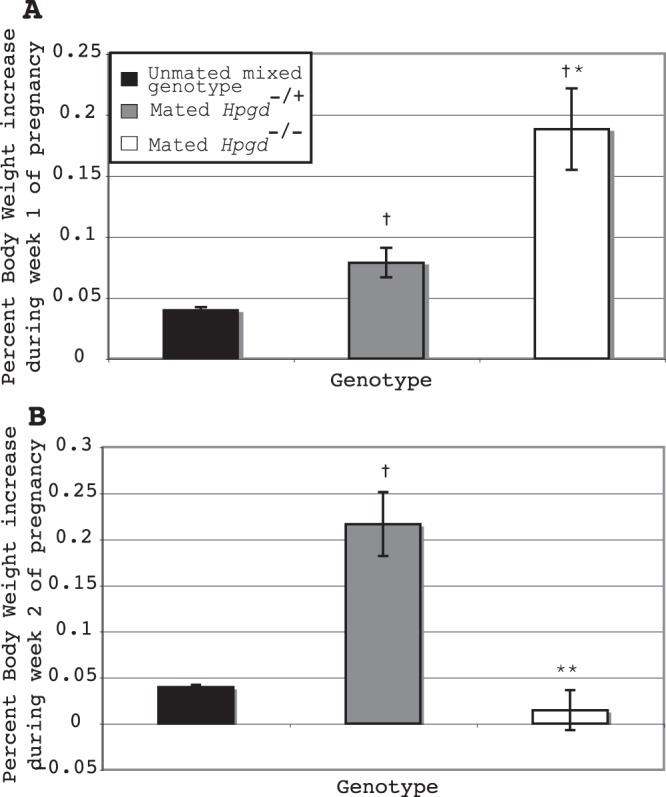
Altered pattern of weight change after mating in 15-HPGD knockout mice. (**A**), Mated *Hpgd*^−/−^ mice have increased weight gain relative to *Hpgd*^+/−^ mice over the first week after mating. (**B**), Mated *Hpgd*^−/−^ mice have decreased weight gain relative to *Hpgd*^+/−^ mice over the second week after mating. †, P < 0.001 relative to unmated mixed genotype, P < 0.001, **P < 0.01 relative to mated *Hpgd*^+/−^, *P < 0.05 relative to mated *Hpgd*^+/−^. Values are mean ± s.e.m. n ≥ 10.

To define the consequences of a complete absence of 15-HPGD activity on birth timing, we mated *Hpgd* knockout mice (*Hpgd*^−/−^ X *Hpgd*^−/−^), *Hpgd* heterozygote mice (*Hpgd*^+/−^ X *Hpgd*^+/−^), or *Hpgd* wild-type mice (*Hpgd*^+/+^ X *Hpgd*^+/+^, Table 2) to compare the duration of pregnancy resulting from variable degrees of HPGD insufficiency. Consistent with what we have reported for hypomorphic mice^7^, in heterozygote matings (*Hpgd*^+/−^ X *Hpgd*^+/−^) labor occurs more than half a day early relative to wild-type matings (*Hpgd*^+/+^ X *Hpgd*^+/+^, Table 2). Surprisingly, *Hpgd* knockout matings (*Hpgd*^−/−^ X *Hpgd*^−/−^) carry no pregnancies to term. To determine when these pregnancies are terminated, we weighed females one week (Fig. 2A) after and two weeks (Fig. 2B) after mating. Interestingly, after mating with *Hpgd*^−/−^ males, *Hpgd*^−/−^ females have increased weight gain relative to *Hpgd*^+/−^ females mated to the same males over the first week of pregnancy, but have decreased weight gain over the second week of pregnancy while the *Hpgd*^+/−^ have accelerated weight gain over this latter time period. These results suggest that knockout pregnancies terminate sometime between the first week and the second week of pregnancy.

**Table 2:** Pregnancy to term in *Hpgd*^−/−^ mice.

Maternal Genotype	Paternal Genotype	n	Percent Pregnant to term	D.P.C. of labor (Mean ± S.E.M.)
*Hpgd*^+/+^	*Hpgd*^+/+^	11	63.6%	19.55 ± 0.04545
*Hpgd*^−/+^	*Hpgd*^−/+^	19	63.2%	18.87 ± 0.1137***
*Hpgd*^−/−^	*Hpgd*^+/+^	8	50%	18.5 ± 0**
*Hpgd*^−/−^	*Hpgd*^−/−^	20	0%***	N.A.

To refine our understanding of when *Hpgd*^−/−^ knockout pregnancies terminate between week one and week two of pregnancy we sacrificed mice on days 5.5–8.5 of pregnancy (Table 3), at this time in pregnancy we found that a smaller percentage of *Hpgd*^−/−^ matings were pregnant than the wild-type matings (17.9% vs 57.9%, Pearson Chi-squared value < 0.01). However, in the mice where pregnancies had been established, *Hpgd*^−/−^ pregnancies look grossly normal on days 5.5–7.5 with the appearance of resorbing implantation sites on day 8.5. These results imply *Hpgd*^−/−^ mice have a deficit in maintaining pregnancy during the first week of pregnancy as well as a deficit in pregnancy maintenance after the first week of pregnancy.

**Figure 3:**
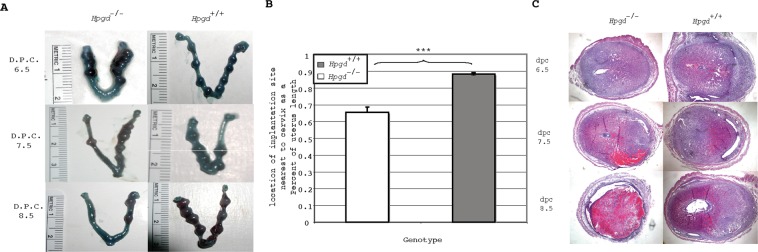
15-HPGD knockout mice have normal appearing pregnancies at dpc 6.5 and 7.5 but lose their pregnancy by d.p.c 8.5. (**A**) Dissected uterus at d.p.c 6.5, 7.5 and 8.5, after perfusion with Chicago Blue Dye. (**B**) Altered implantation spacing defined by location of implantation site in closest proximity to the cervix expressed as a function of percentage of uterine horn length. ***P < 0.001. Values are mean ± s.e.m. n ≥ 10. (**C**) The histology of *Hpgd*^−/−^ implantation sites appears normal until day 8.5; Hemetoxylin and Eosin stain of implantation site sections on dpc 6.5, 7.5 and 8.5.

To examine the possibility that decidual vascularization was defective in these mice, we compared the vascularization of implantation sites between knockout matings and wild-type matings using Chicago Blue Dye on days 6.5, 7.5 and 8.5 (Fig. 3A). The decidual vascularization was similar between intact pregnancies in both genotypes, however, we noticed an alteration in implantation spacing in the knockout mice. Specifically, while the wild-type mice generally had an implantation site in close proximity to the cervix, knockout matings exhibit crowding of implantation sites near the distal end of the uterus and lack implantation sites near the cervix. To quantify this altered implantation spacing we compared the location of the implantation site nearest the cervix as a percent of the total uterine horn length between knockout pregnancies and wild-type pregnancies. It was found that the implantation site nearest the cervix was statistically significantly closer to the cervix in wild-type pregnancies than in knockout pregnancies, however, the physiological significance of this observation remains unknown.

In addition to examining implantation site spacing, we also examined the implantation sites histologically (Fig. 3C). On days 6.5 and 7.5 the knockout implantation sites look entirely normal, however, by day 8.5 of pregnancy, the knockout implantation sites universally appear necrotic with inflammatory infiltrates.

**Figure 4:**
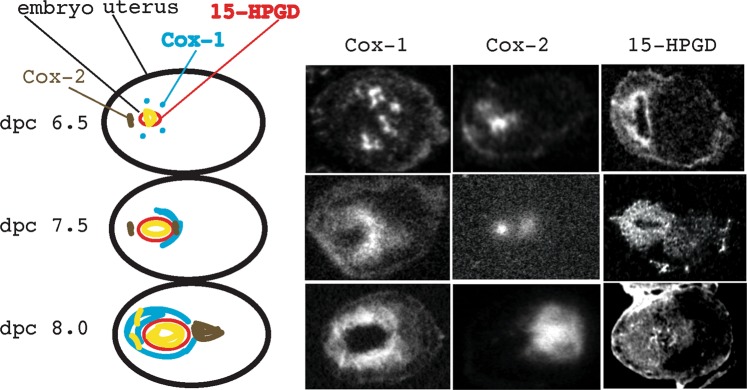
15-HPGD and Cycloxygenases 1 and 2 exhibit specific patterns in the wild-type implantation site at the time of pregnancy loss in the 15-HPGD knockout mice (dpc 6.5–8.0). In the left column is a schematic representation of the characteristic expression of Cox-1 (blue), Cox-2 (brown) and 15-HPGD (red) at days 6.5, 7.5 and 8.0. In the second column, is the characteristic expression of Cox-1, then the characteristic expression pattern of Cox-2 and then the characteristic expression pattern of 15-HPGD. Typical sections from different embryos shown, n = 2–3 for each.

Since PGE_2_ and PGF2α are generated by the cycloxygenases (Cox-1 and Cox-2) and broken down by 15-HPGD, we sought to examine the mRNA expression patterns of these three enzymes in implantation sites using *in situ* hybridization (Fig. 4). The pattern of Cox-1 and Cox-2 mRNA expression is similar between genotypes until day 8.5 and is consistent with results reported previously^22^.

Specifically, Cox-1 was observed in several distinct locations on day 6.5, then observed surrounding the developing blastocyst in secondary decidual cells at both the mesometrial and antimesometrial poles on day 7.5 and similarly localized with increasing intensity on dpc 8.0. While Cox-2 was observed on dpc 6.5 around the blastocyst at the antimesometrial pole and then in the decidualizing stromal cells on dpc 8.0 at the mesometrial pole of the implantation site.

Over this same period 15-HPGD exhibits a characteristic expression pattern, expressed on dpc 6.5, 7.5 and 8.0 at the edges of the developing embryo. These results suggest that the expression of 15-HPGD occurs in the embryo at dpc 6.5, 7.5 and 8.0 to metabolize maternally generated PGs and prevent fetal resorption.

**Figure 5:**
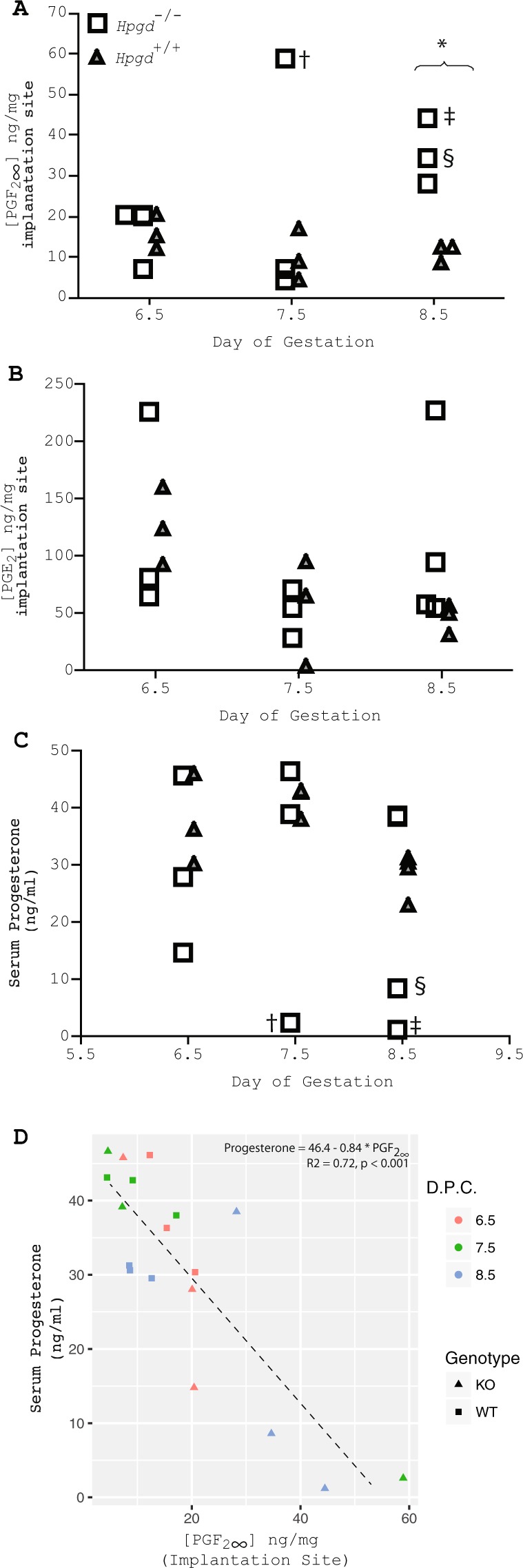
15-HPGD knockout mice have normal levels of PGE2 but increased levels of PGF2α and decreasing levels of serum progesterone at the time of pregnancy loss. (**A**) implantation site PGF_2α_ concentrations in the wild type (triangle) and the KO at dpc 6.5, 7.5 and 8.0. *P < 0.01. (**B**) implantation site PGF_2α_ concentrations in the wild type (triangle) and the KO at dpc 6.5, 7.5 and 8.0. (**C**) serum progesterone concentrations in the wild type (triangle) and the KO at dpc 6.5, 7.5 and 8.0%, # designate samples from the same mouse in (**A–C**). n = 3 (**D**) serum progesterone concentrations correlate inversely with local PGF_2α_ concentrations in the wild type (square) and the KO (triangle) at dpc 6.5 (red), 7.5 (green) and 8.0 (blue), n = 3.

After establishing that the Cox enzymes are expressed on dpc 6.5–8.0 of implantation, we examined concentrations of PGE_2_ and PGF_2α_ in implantation sites on dpc 6.5, 7.5 and 8.5 (Fig. 5A,B). We found no statistically significant difference between implantation site PGE_2_ concentrations on any of these dpc (Fig. 5B). However, we observed a consistent increase in implantation site PGF_2α_ concentrations on dpc 8.0 in *Hpgd*^−/−^ pregnancies. In some pregnancies this elevation occurs on dpc 7.5 (Fig. 5A). Because PGF_2α_ is luteolytic we compared serum progesterone concentrations between *Hpgd*^−/−^ pregnancies and *Hpgd*^+/+^ pregnancies over this same time period (Fig. 5C). Serum progesterone decrease appears to closely follow PGF_2α_ elevation (Fig. 5D); that is, in the *Hpgd*^−/−^ pregnancies with the most elevated implantation site PGF_2α_ at dpc 7.5 and 8.5 progesteone concentrations have decreased to non-pregnant levels, while in the pregnancy with the least elevated PGF_2α_ concentrations on dpc 8.5, serum progesterone concentrations are similar to those in *Hpgd*^+/+^ pregnancies (Fig. 5D).

To determine if the pregnancy loss in *Hpgd*^−/−^ matings is due to the combined effects of maternal and fetal 15-HPGD deficiency or due to the absence of 15-HPGD activity in all of the fetuses, we performed embryo transfer experiments transferring either *Hpgd*^+/+^ or *Hpgd*^−/−^ embryos into *Hpgd*^+/+^ mothers (Table 4). No statistically significant difference was observed between genotypes in the ability to mate after superovulation or in the number of embryos produced after superovulation (data not shown). Surprisingly, while *Hpgd*^+/+^ embryos are able to be transferred, implant and make it to term when transferred into *Hpgd*^+/+^ pseudopregnant females at a rate comprable to normal pregnancy, *Hpgd*^−/−^ embryos are unable to implant and be carried to term when transferred into these same *Hpgd*^+/+^ pseudopregnant females.

**Table 3:** Pregnancy to dpc 5.5–8.5 in Gestation in *Hpgd*^−/−^ mice.

Genotype of Parents	n	% pregnant
*Hpgd*^+/+^	38	57.9%
*Hpgd*^−/−^	28	17.9.%**

**Pearson Chi-squared value < 0.01.

Given the role of an acute rise in locally produced PGs in the pregnancy loss observed in KO pregnancies we wished to examine whether short term treatment with cycloxygenase inhibitors could rescue the phenotype of pregnancy loss. To answer this question, we treated mated KO females (*Hpgd*^−/−^ X *Hpgd*^−/−^) in the evening of dpc 6.5, the morning and evening of dpc 7.5 and the morning of dpc 8.5 with 5 mg/kg of indomethacin by oral gavage. While indomethacin treatment increased the rate of pregnancy maintenance to day 8.5 from 0% (n = 12) to 62.5% (n = 8, Pearson Chi-squared value < 0.05), it did not allow the maintenance of pregnancy to term (n = 7). Additionally, four of the seven mice gavaged, became visibly ill between dpc 9.5 and 10. Three of these four mice, all of whom were pregnant with normal appearing implantation sites on autopsy, died between dpc 10 and 10.5.

## Discussion

We had previously described early labor in the context of 15-HPGD hypomorphic mice^7^; the early labor observed in 15-HPGD heterzygote pregnancies (*Hpgd*^−/+^ X *Hpgd*^−/+^) is consistent with what we have observed in 15-HPGD hypomorph pregnancies (*Hpgd*^H/H^ X *Hpgd*^H/H^). However, the pregnancy loss observed in KO pregnancies (*Hpgd*^−/−^ X *Hpgd*^−/−^) is a novel and unexpected finding. Given the role PGE_2_ plays in ovulation we might have expected an alteration in the catabolism of this prostaglandin might alter this process. However, the pregnancy loss observed in KO pregnancies appears unrelated to any alteration in ovulation. Interestingly, pregnancy resorption does not occur in matings between wild-type males and KO females (Table 2) suggesting that maternal 15-HPGD is dispensable for maintenance of pregnancy in the context of fetal 15-HPGD activity. Our indomethacin rescue experiments reveal that pregnancy loss prior to dpc 8.5 can be rescued by indomethacin gavage. However, pregnancies rescued to dpc 8.5 are not maintained to term and mortality occurs in a significant number of females after pregnancy rescue at dpc 10.5. Maternal health is adversely affected either when indomethacin is used to prolong pregnancy or when pre-term pregnancy termination occurs. Early pregnancy resorption avoids these effects. Thus, it appears that resorption of pregnancies by dpc 8.5 protects pregnant KO mothers from later mortality due to altered physiology.

Our embryo transfer experiments reveal that pregnancies comprised entirely of *Hpgd*^−/−^ fetuses are unable to be maintained to term. Such a result is particularly surprising given the ability of *Hpgd*^−/−^ fetuses to survive to term in Mendelian ratios in the context of 15-HPGD heterzygote pregnancy (*Hpgd*^+/−^ X *Hpgd*^+/−^). Thus it is likely that this pregnancy resorption in pregnancies with only KO fetuses represents not a defect in development per se, but a defect in signaling from the fetuses to the mother. This signal is particularly unique in that it is not an individual signal, but a collective signal. Such a signal would be unique in that it could be rescued by the presence of 15-HPGD activity in another fetus.

Previous work has delineated the roles of Cox-1 and Cox-2 in fertility and we have previously identified a role for 15-HPGD in parturition^3,22–24^. This work reveals a previously undescribed role in the maintenance of pregnancy for 15-HPGD.

In summary, we demonstrate a previously unappreciated role of HPGD activity in the maintenance of pregnancy in the mouse. Moreover, HPGD activity in the fetus is essential to prevent early termination of pregnancy and maternal morbidity. These results may also have important implications for pregnancy loss in humans. More than 50% of all pregnancies are lost prior to week six of gestation^25,26^, and up to 5% of couples attempting to conceive have recurrent unexplained early pregnancy loss^27^. Our results suggest that altered 15-HPGD function may be the cause of some of this unexplained early pregnancy loss. These studies reinforce the primacy of prostaglandin signaling as a key evolutionary component for viviparity, and suggest new targets for investigation in early human pregnancy loss.

**Table 4:** Pregnancy to term after Embryo Transfer in *Hpgd*^−/−^ mice.

Maternal genotype	Embryo genotype	n	Percent Pregnant to term	D.P.C. of labor (Mean ± S.E.M.)
*Hpgd* ^+/+^	*Hpgd* ^+/+^	9	66.7%	19.5 ± 0
*Hpgd* ^+/+^	*Hpgd* ^−/−^	7	0%**	NA

## Methods

### Generation of Hpgd^−/−^ mice

To construct the conditional 15-HPGD targeting vector, loxP sites were inserted into unique restriction sites upstream of exon 1 (HindIII) and downstream of exon 2 (BamHI) in the *Hpgd* gene. The downstream loxP site was adjacent to a neomycin selection cassette. To obtain embryonic stem (ES) cell clones heterozygous for a targeting event at the *Hpgd* locus the linearized construct was electroporated into TC1 ES cells^28^. Clones selected for G418 resistance were analyzed by Southern blot for homologous recombination into the endogenous locus. ES clones containing the targeting construct integrated into the endogenous locus were injected into C57BL/6 blastocysts to generate high percentage male chimeras. Using this method a conditional KO mouse line was created. All further mice generated were of a mixed C57BL/6–129/SvJ background, with littermates used as controls. To generate heterozygote conventional knockout mice (*Hpgd*^−/+^) from the conditional knockout line (*Hpgd*^LOXP/LOXP^), conditional knockout mice were mated to actin-Cre mice and then mated to achieve the desired genotype (*Hpgd*^+/+^, *Hpgd*^−/+^, *Hpgd*^−/−^). To survive to two weeks, all conventional knockout mice (*15-Hpgd*^−/−^) required indomethacin injection at birth as described previously^21^.

### Labor timing and pregnancy

To examine the effect of a reduction in 15-HPGD activity on the timing of labor, female mice were placed in cages with a male, removed from that cage after mating (determined by detection of a copulation plug) and observed for the timing of birth of the first pup twice daily. Results were analyzed using a simple t test. Term refers to the time of birth of pups. We followed all mice until 21.5 days post copulation.

### Hormone measurements

Plasma progesterone concentration was determined by radioimmunoassay kit as described by the manufacturer (Diagnostic Products, Los Angeles) on blood samples obtained by retroorbital phlebotomy. Each measurement was performed on at least 3 separate pregnancies of each genotpe and analyzed for significance by two-way ANOVA, and then each pair was analyzed by Tukey post-tests. Uterine, placental, fetal membrane and ovarian PGF2α and PGE2 were measured on tissue harvested and rapidly frozen in liquid nitrogen from three separate pregnancies per genotype at each time point. Tissue was weighed while frozen and then homogenized in 100% ethanol for extraction of prostaglandins. Debris was removed by centrifugation and each supernatant was assayed in duplicate per the manufacturer’s instructions (Oxford Biomedical Research, Oxford MI). Measurements were analyzed for statistical significance by Students T-test.

### Northern hybridization analyses

RNA was prepared from frozen tissue using the RNEasy Midi kit (Qiagen) as described by the manufacturer. Five micrograms of total RNA from each tissue and genotype were subjected to electrophoresis through 1.7% agarose-formaldehyde gels and transferred to nitrocellulose membranes. cRNA probes specific for mouse 15-HPGD mRNA (834-bp fragment from nucleotide 13 to 846 in pBluescript SK II+) labeled with [α−32 P]-UTP were generated by transcription with T3 polymerase and hybridized at 65 °C in 50% formamide-containing buffer. After washing, hybridizing probes were quantitated on a Molecular Dynamics PhosphorImager. Each mRNA hybridization signal was corrected for loading and recovery by normalization to cyclophilin A mRNA hybridization on the same filter. Statistical significance was determined by ANOVA.

### *In situ* hybridization

*In situ* hybridization was performed as described previously^29^. Fetuses with surrounding membranes and uteri were fixed by immersion in 4% paraformaldehyde in PBS for 24 h at 4 °C. Samples were then cryopreserved in 10% sucrose in PBS and embedded in OCT compound (Sakura Finetek, Torrance, CA) for sectioning on a cryostat. Ten-micrometer sections were thaw mounted onto Superfrost plus slides (Fisher Scientific), and hybridized to an [α −33P]-UTP-labeled 15-HPGD antisense riboprobe. After washing, slides exposed to autoradiographic film and scanned at high resolution. In Fig. 4 typical non-consecutive sections from different embryos are shown.

### 15-HPGD activity measurements

15-HPGH activity was assayed as described previously^30^ by measuring the transfer of tritium from 15(S)-[15–3 H]-PGE2 to glutamate by coupling 15-HPGD with glutamate dehydrogenase. Results were analyzed for statistical significance by ANOVA.

### Embryo transfer experiments

3 week old females superovulated and mated. Simultaneously, 8 week old females were mated with vasectomized males. Both sets of females are checked for copulation plugs the morning after mating (dpc 0.5). 2.5 days after mating, embryos are harvested from the 3 week old females and 20–25 are injected into the uterus of 8 week old pseudopregnant females.

### Gavage of KO matings

Pregnant females were gavaged using indomethacin in PBS (with 1% tween 20).

### Ethical approval/experimental animals

All animal experimentation described was conducted in accord with accepted standards of humane animal care, and was approved by the Washington University in St. Louis Animal Studies Committee.

## Data Availability

The datasets generated during and/or analysed during the current study are available from the corresponding author on reasonable request.
